# Systematic construction and validation of an epithelial–mesenchymal transition risk model to predict prognosis of lung adenocarcinoma

**DOI:** 10.18632/aging.202186

**Published:** 2020-12-03

**Authors:** Yunliang Tang, Yanxia Jiang, Cheng Qing, Jiao Wang, Zhenguo Zeng

**Affiliations:** 1 Department of Critical Care Medicine, First Affiliated Hospital of Nanchang University, Nanchang 330006, Jiangxi, China; 2 Department of Rehabilitation Medicine, First Affiliated Hospital of Nanchang University, Nanchang 330006, Jiangxi, China; 3 Department of Endocrinology and Metabolism, First Affiliated Hospital of Nanchang University, Nanchang 330006, Jiangxi, China

**Keywords:** epithelial-mesenchymal transition, lung adenocarcinoma, gene signature, nomogram, prognosis

## Abstract

Epithelial–mesenchymal transition (EMT) has been shown to be linked to a poor prognosis, particularly in patients with non-small-cell lung cancer. Nevertheless, little is known regarding the existence of EMT-related gene signatures and their prognostic values in lung adenocarcinoma (LUAD). In the current study, we systematically profiled the mRNA expression data of patients with LUAD in The Cancer Genome Atlas and Gene Expression Omnibus databases using a total of 1,184 EMT-related genes. The prognostic values of the EMT-related genes used to develop risk score models for overall survival were determined using LASSO and Cox regression analyses. A prognostic signature that consisted of nine unique EMT-related genes was generated using a training set. A nomogram, incorporating this EMT-related gene signature and clinical features of patients with LUAD, was constructed for potential clinical use. Calibration plots, decision-making curves, and receiver operating characteristic curve analysis showed that this model had a good ability to predict the survival of patients with LUAD. The EMT-associated gene signature and prognostic nomogram established in this study were reliable in predicting the survival of patients with LUAD. Thus, we first identified a novel EMT-related gene signature and developed a nomogram for predicting the prognosis of patients with LUAD.

## INTRODUCTION

Lung cancer, particularly non-small-cell lung cancer (NSCLC) and small-cell lung cancer, is a malignant tumor with the highest rate of cancer-related mortality worldwide [1]. NSCLC accounts for 80–85% of cases of diagnosed lung cancer and can be pathologically divided into three types, namely, adenocarcinoma, squamous cell carcinoma, and large-cell carcinoma [2]. Among these subtypes, lung adenocarcinoma (LUAD) is the most prevalent, accounting for approximately 40% of all lung tumors [3]. However, it has been reported that LUAD is a highly heterogeneous and aggressive disease, which is frequently associated with genetic alterations, including *TP53*, Kirsten rat sarcoma viral oncogene homolog (*KRAS*), and epidermal growth factor receptor (*EGFR*) gene mutations, as well as the anaplastic lymphoma kinase (*ALK*)–*NPM* fusion [4]. Advances in chemotherapy, radiation therapy, and targeted therapy have reduced mortality among patients with LUAD over the years; however, the long-term survival rates have barely improved, especially compared with those of other cancers [5]. Thus, it is imperative to explore the molecular mechanisms of LUAD progression and develop better tumor prognostic markers that accurately predict the survival of patients with LUAD [6, 7].

Epithelial–mesenchymal transition (EMT) is a biological process whereby epithelial cells acquire a mesenchymal phenotype. During EMT, epithelial cells lose their adhesiveness, secrete enzymes that dissolve the extracellular matrix, and acquire migratory ability [8]. Recently, an increasing number of studies have demonstrated that EMT leads to tumor metastasis and is an important process by which more than 90% of malignant epithelial cells become involved in carcinogenesis in adults [9–11]. Furthermore, a number of studies have reported that EMT is a marker of poor prognosis in patients with LUAD [12–15]. However, the information on the prognostic significance of EMT-related genes (ERGs) and their biological function in LUAD remains rudimentary and inconclusive.

Therefore, the development of new biomarkers, based on ERG signatures, may optimize the selection of patients at the highest risk of mortality and provide novel insights into gene-targeting therapy. To this end, this study aimed to identify an ERG signature that could predict the prognosis of patients with LUAD with high accuracy.

## RESULTS

### Development of an ERG signature using a training set

**Figure 1:**
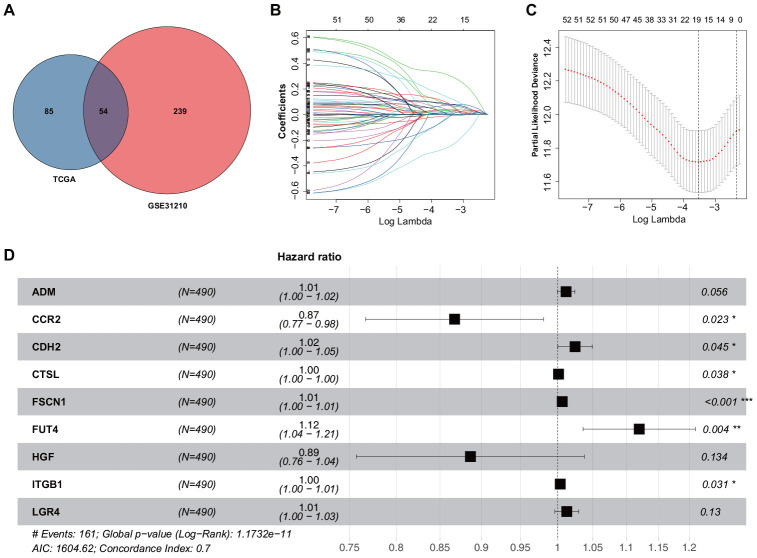
**Construction of the ERG signature.** (**A**) Identification of ERGs correlated with survival. (**B**) LASSO coefficients of the ERGs. Each curve represents an ERG. (**C**) Cross-validation of gene selection using 1-SE criteria in the LASSO regression analysis. (**D**) Forest plot of multivariate Cox regression analysis.

To examine the correlation of gene expression profiles with EMT scores, the expression data for ERGs and corresponding survival time were subjected to univariate Cox regression analysis. Eventually, 139 and 293 ERGs were found to be significantly correlated with the prognosis of patients based on The Cancer Genome Atlas (TCGA) and GSE31210 datasets, respectively (*P* < 0.05; Supplementary Tables 1, 2). Thereafter, 54 prognostic ERGs that overlapped between the two datasets were used to develop a prognostic gene signature (Figure 1A). Using LASSO-penalized Cox regression analysis of the training set, we identified 19 genes (Figure 1B, 1C). Subsequently, multivariate Cox regression analysis was used to establish the ERG signature, and nine genes were finally selected as predictors of overall survival (OS) in patients with LUAD (Figure 1D).

### ERG expression and alterations in LUAD

**Figure 2:**
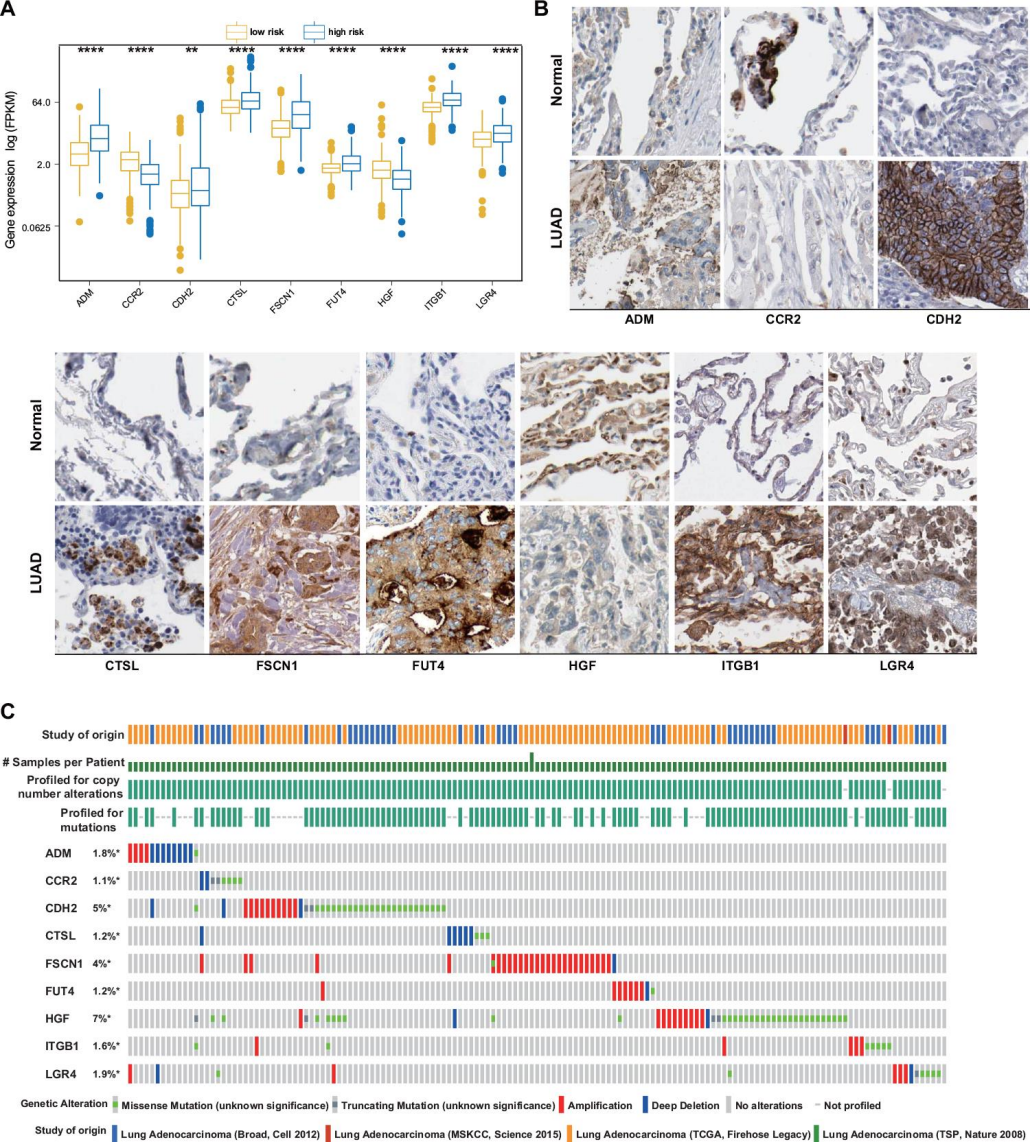
**ERG expression and mutation landscape of LUAD.** (**A**) Comparison of mRNA expression levels between LUAD and normal tissues in the training set. (**B**) Comparison of protein levels between LUAD and normal tissues in the HPA. (**C**) Mutational profiles of LUAD (obtained from the cBioPortal for Cancer Genomics).

The mRNA expression levels of the nine identified ERGs that were selected as the LUAD signature were examined using the training set. It was found that in the LUAD tissue samples, the adrenomedullin (*ADM*), cadherin 2 (*CDH2*), cathepsin L (*CTSL*), fascin-1 (*FSCN1*), fucosyltransferase 4 (*FUT4*), integrin beta-1 (*ITGB1*), and leucine-rich repeat-containing G-protein-coupled receptor 4 (*LGR4*) gene expression levels were significantly higher and the C-C chemokine receptor 2 (*CCR2)* and hepatocyte growth factor (*HGF)* gene expression levels were significantly lower than those in the normal lung tissues (Figure 2A). To further validate the data, the corresponding protein expression levels were analyzed using the Human Protein Atlas (HPA) database. It was found that the protein levels matched the mRNA expression levels of these ERGs (Figure 2B).

The presence of potential genetic alterations in our signature was explored in patients with LUAD using the cBioPortal database, including four LUAD datasets. Among these datasets, the rates of gene alterations, including missense and truncating mutations, amplifications, and deep deletions, ranged from 1.1% to 7%, with amplifications being the most commonly observed alterations (Figure 2C).

### Gene set enrichment analysis (GSEA) and gene set variation analysis (GSVA)

**Figure 3:**
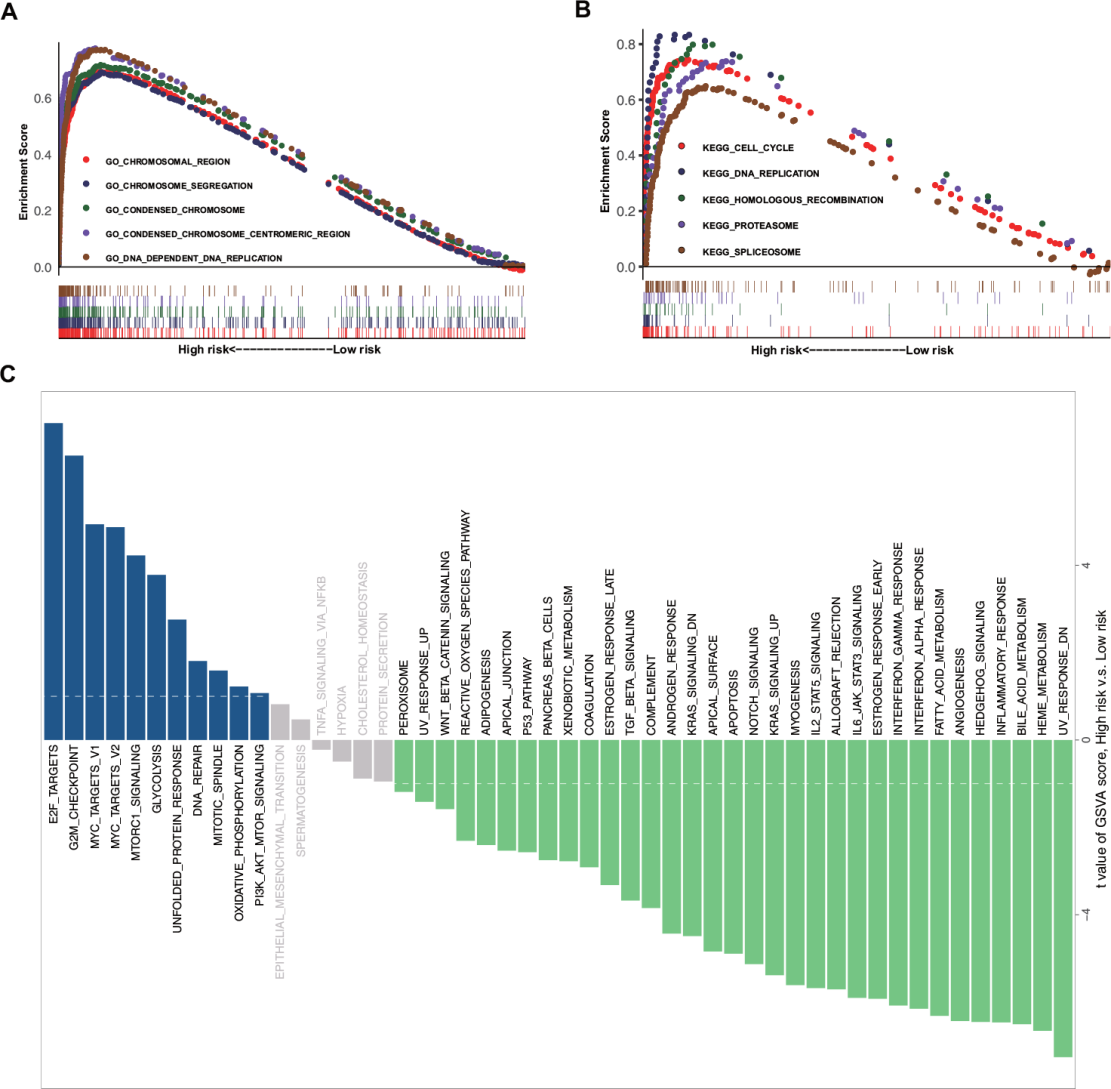
**GSEA and GSVA data.** (**A**) Top five representative GO annotation terms in the high-risk group. (**B**) Top five representative KEGG pathways in the high-risk group. (**C**) Comparison of the low- and high-risk groups using GSVA.

Further, we investigated and compared the potential functional mechanisms between high- and low-risk groups. GSEA was conducted on the training set, and a number of enriched terms were observed in the high-risk group, most of which were cancer-related biological functions and signaling pathways. The top five Gene Ontology (GO) biological processes and Kyoto Encyclopedia of Genes and Genomes (KEGG) pathways, which include the chromosomal region, chromosome segregation, condensed chromosome, cell cycle, DNA replication, and homologous recombination, are shown in Figure 3A, 3B. The GSVA results demonstrated that the EMT-related signaling pathways, such as E2F targets, G2M checkpoint, MYC targets v1, MYC targets v2, and Mtorc1 signaling, were significantly activated in the high-risk group (Figure 3C).

### Prognostic value of the ERG signature in the training set

**Figure 4:**
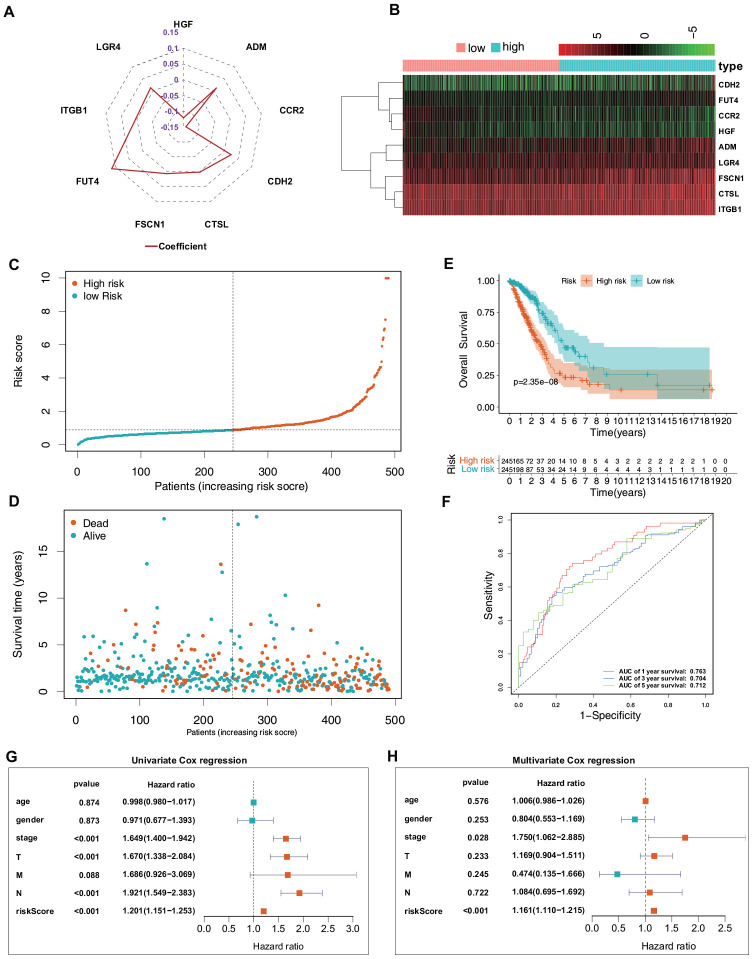
**Predictive value of the ERG signature for LUAD in the training set (TCGA).** (**A**) Value of each coefficient representing its relative contribution to the predictive signature. (**B**) Heatmap of the mRNA expression levels of the nine signature-comprising ERGs. (**C**) Rank of risk signature and score distribution. (**D**) Distribution of patients in the low- and high-risk groups based on their survival status (OS). (**E**) OS times of the patients between high- and low-risk groups. (**F**) Time-dependent ROC curve analysis for the prediction of 1-, 3-, and 5-year OS using the ERG signature. (**G**) Univariate Cox regression analysis of the ERG signature and clinical features of the patients. (**H**) Multivariate Cox regression analysis of the ERG signature and clinical features of the patients.

Based on the median risk score, the included 490 patients with LUAD from TCGA were equally divided into low-risk (n = 245) and high-risk (n = 245) groups. Figure 4A shows the coefficient of the formula and the risk scores [risk scores = (0.011794409 × *ADM* mRNA level) + (−0.142467012 × *CCR2* mRNA level) + (0.024230703 × *CDH2* mRNA level) + (0.001349971 × *CTSL* mRNA level) + (0.006464507 × *FSCN1* mRNA level) + (0.113002543 × *FUT4* mRNA level) + (−0.120528327 × *HGF* mRNA level) + (0.003726199 × *ITGB1* mRNA level) + (0.012745463 × *LGR4* mRNA level)]. Figure 4B–4D shows a heatmap of the expression profiles of the nine ERGs, the risk score distribution, and the vital statuses of the patients with LUAD from the high- and low-risk groups. Kaplan–Meier survival curves exhibited a significantly worse OS in the high-risk than in the low-risk group (*P* = 2.35e−8; Figure 4E). Next, receiver operating characteristic (ROC) curve analysis was carried out to assess the discrimination capacity of the nine-gene signature. The areas under the curves (AUCs) for 1-, 3-, and 5-year OS predictions were 0.763, 0.704, and 0.712, respectively (Figure 4F), which indicated a good performance of this nine-ERG signature for prognostic prediction. Univariate and multivariate Cox regression analyses suggested that the ERG signature could be regarded as an independent predictor of OS after adjustment for demographic and clinical features, including age, sex, tumor grade, and TNM stage (Figure 4G, 4H).

### Validation of the ERG signature

**Figure 5:**
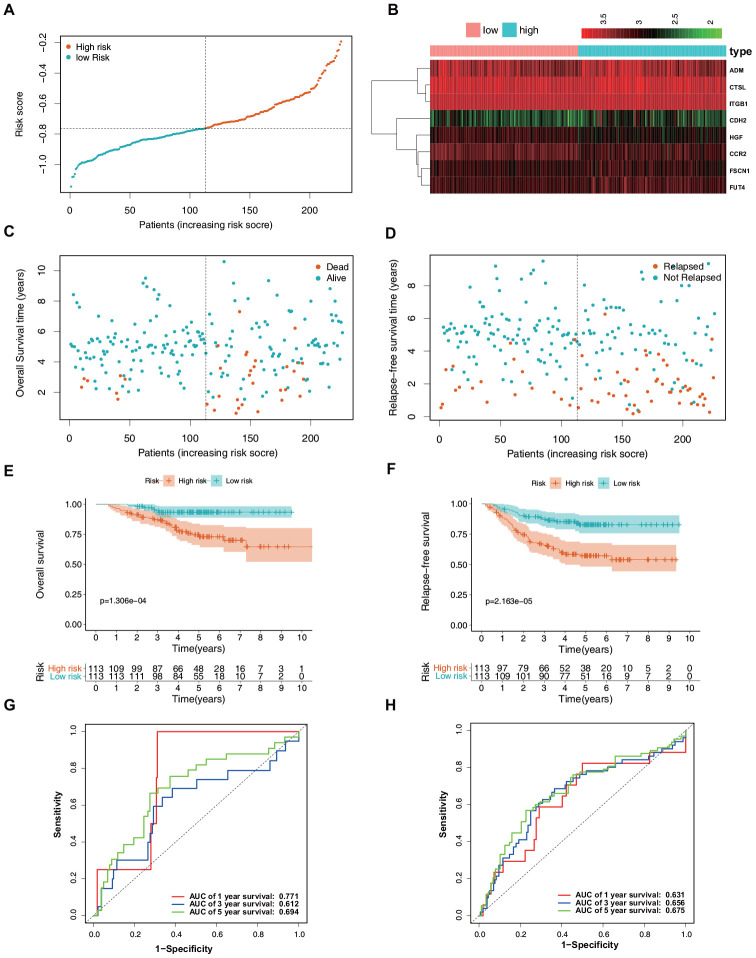
**Verification of the predictive value of the ERG signature for LUAD in the validation set (GSE31210).** (**A**) Risk signature rank and score distribution. (**B**) Heatmap of the mRNA expression levels of the nine genes included in the signature. (**C**) Distribution of patients in the high- and low-risk groups based on their survival status (OS). (**D**) Distribution of patients in the high- and low-risk groups based on their survival status (RFS). (**E**) OS of the patients in the low- and high-risk groups. (**F**) RFS of the patients in the low- and high-risk groups. (**G**) Time-dependent ROC curve analysis for the prediction of 1-, 3-, and 5-year OS using the ERG signature. (**H**) Time-dependent ROC curve analysis for the prediction of 1-, 3-, and 5-year RFS using the ERG signature.

To validate the reliability of the ERG signature, the risk score was calculated for each patient in the validation set (GSE31210) using the same formula that was used for the patients from TCGA. The samples were then separated into high- and low-risk groups based on the median value of the risk scores. In the validation set, the distribution of the risk scores, survival statuses of the patients, and a heatmap of expression profiles of the nine ERGs (Figure 5A–5D) showed similar trends to those in the training set. Further survival analysis indicated that the high-risk patients had a significantly worse OS and relapse-free survival (RFS) than did the low-risk patients (Figure 5E, 5F). Time-dependent ROC curves were generated to explore the prognostic values of the nine ERG-based risk scores. It was found that the ERG signature could accurately predict the prognosis of the patients (Figure 5G, 5H). Taken together, the ERG signature was capable of predicting the survival of patients with LUAD.

### Correlation between the ERG signature and clinical and demographic features of patients

**Figure 6:**
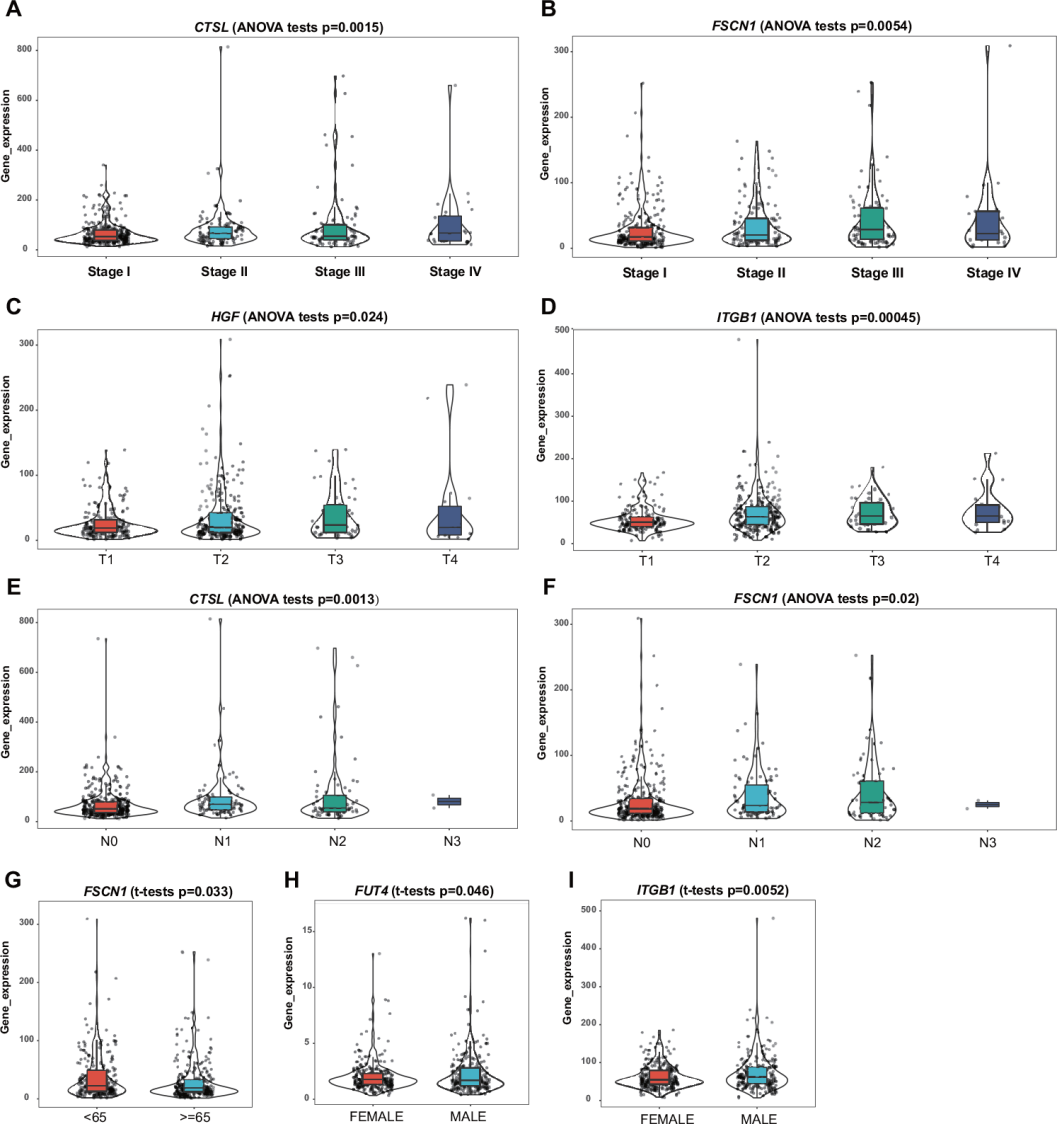
**Correlation of the mRNA expression levels of the prognostic ERG signature with demographic and clinicopathological characteristics of patients with LUAD.** (**A**) *CTSL* and TNM stage. (**B**) *FSCN1* and TNM stage. (**C**) *HGF* and T stage. (**D**) *ITGB1* and T stage. (**E**) *CTSL* and N stage. (**F**) *FSCN1* and N stage. (**G**) *FSCN1* and age. (**H**) *FUT4* and sex. (**I**) *ITGB1* and sex.

Clinical and demographic features, including age, sex, pathological TNM stage, and pathological tumor stage, were analyzed in the training set, and the relationships between the screened genes and clinical indexes were explored. The results suggested a differential expression of *CTSL*, *FSCN1*, *HGF*, *ITGB1*, and *FUT4* in patients with various clinical features (Figure 6A–6I).

### Subgroup analysis of the ERG signature

**Figure 7:**
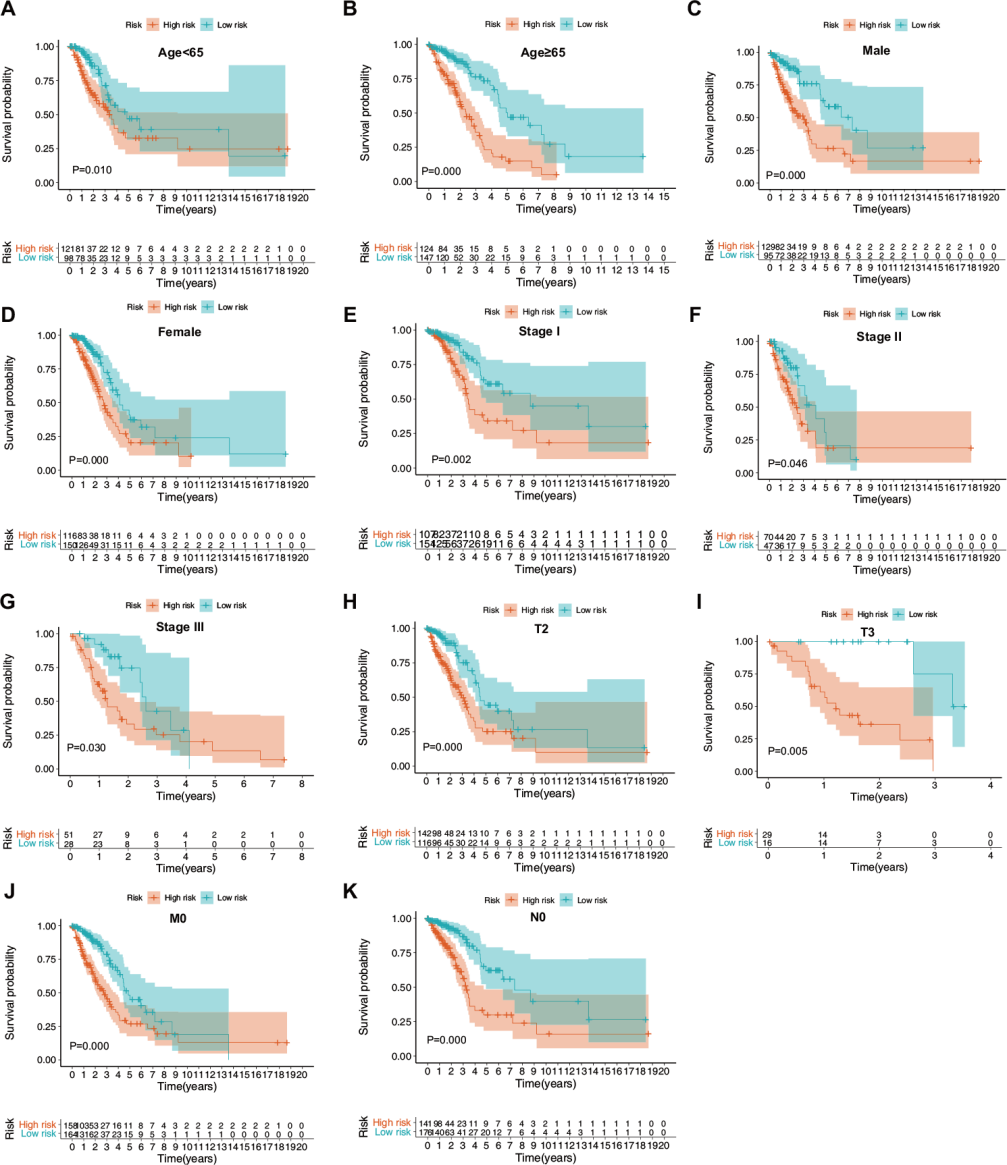
**Confirmation of the ERG signature via stratification of patients from the training set based on specific demographic and clinical features.** (**A**) Age < 65 years; (**B**) age ≥ 65 years; (**C**) male; (**D**) female; (**E**) stage I; (**F**) stage II; (**G**) stage III; (**H**) T2; (**I**) T3; (**J**) M0; and (**K**) N0.

The prognostic value of the ERG signature was further explored in subgroups of patients with LUAD with various clinical and demographic features. Stratification analysis was carried out according to the clinical and demographic features, including age, sex, T stage, N stage, M stage, and pathological tumor stage (Figure 7A–7K). It was found that the ERG signature was useful in most of the subgroups (Tables 1, 2).

**Table 1:** Association between the ERG signature and OS of patients with LUAD in the training set (TCGA, n = 490), stratified by demographic and clinical characteristics.

**Characteristics**	**Number (high-/low-risk group)**	**Proportion of patients**	**HR (95% CI)**	***P*-value**
**Age (years)**				
≥ 65	124/147	55.3%	0.554 (0.445–0.688)	0.000
< 65	121/98	44.7%	0.509 (0.306–0.849)	0.010
**Sex**
Female	116/150	54.3%	0.450 (0.290–0.698)	0.000
Male	129/95	45.7%	0.592 (0.457–0.765)	0.000
**Stage**
I	107/154	53.3%	0.654 (0.499–0.858)	0.002
II	70/47	23.9%	0.731 (0.537–0.995)	0.046
III	51/28	16.1%	0.673 (0.470–0.962)	0.030
IV	16/9	5.1%	0.611 (0.322–1.159)	0.132
NA	1/7	1.6%	−	−
**T stage**
T1	63/103	21.6%	0.744 (0.545–1.016)	0.063
T2	142/116	52.7%	0.667 (0.534–0.833)	0.000
T3	29/16	9.2%	0.233 (0.084–0.643)	0.005
T4	10/8	3.7%	0.500 (0.224–1.114)	0.090
NA	½	0.6%	−	−
**M stage**
M0	158/164	65.7%	0.644 (0.527–0.787)	0.000
M1	16/8	4.9%	0.518 (0.243–1.103)	0.088
NA	71/73	29.4%	−	−
**N stage**
N0	141/176	64.7%	0.589 (0.463–0.749)	0.000
N1	57/35	18.8%	0.811 (0.600–1.096)	0.173
N2	45/23	13.9%	0.777 (0.531–1.138)	0.195
N3	0/2	0.4%	−	−
NA	2/9	2.2%	−	−

NA, not available; HR, hazard ratio; CI, confidence interval.

**Table 2:** Association between the ERG signature and survival of patients with LUAD in the validation set (GSE31210, n=226), stratified by demographic and clinical characteristics.

**Characteristics**	**Number (high-/low-risk group)**	**Proportion of patients**	**OS**		**RFS**	
			**HR (95% CI)**	***P*-value**	**HR (95% CI)**	***P*-value**
**Age (years)**						
≥ 65	87/77	72.6%	0.643 (0.371−1.112)	0.114	0.666 (0.429−1.005)	0.053
< 65	26/36	27.4%	0.103 (0.024−0.442)	0.002	0.504 (0.348−0.729)	0.000
**Sex**						
Female	56/65	53.5%	0.300 (0.096−0.937)	0.038	0.348 (0.165−0.737)	0.006
Male	57/48	46.5%	0.437 (0.236−0.810)	0.009	0.559 (0.374−0.834)	0.004
**Smoking status**						
Ever smoker	61/50	49.1%	0.434 (0.235−0.802)	0.008	0.506 (0.334−0.767)	0.001
Never smoker	52/63	50.9%	0.556 (0.313−0.988)	0.046	0.647 (0.446−0.940)	0.022
**Stage**						
I	75/93	74.3%	0.415 (0.222−0.775)	0.006	0.518 (0.359−0.741)	0.000
II	38/20	25.7%	0.663 (0.380−1.158)	0.148	0.812 (0.537−1.228)	0.323
**Mutation**						
*ALK* fusion	9/2	4.9%	0.189 (0.000−377.477)	0.668	0.187 (0.000−313.329)	0.658
*EGFR* mutation	37/31	30.1%	0.496 (0.264−0.932)	0.029	0.638 (0.420−0.968)	0.035
*KRAS* mutation	52/75	56.2%	0.523 (0.292−0.934)	0.029	0.558 (0.381−0.815)	0.003
Wild-type *EGFR/KRAS/ALK*	15/5	8.8%	0.175 (0.001−41.803)	0.532	0.172 (0.004−7.474)	0.360

HR, hazard ratio; CI, confidence interval.

### Comparison of immune cell types between the low- and high-risk groups

**Figure 8:**
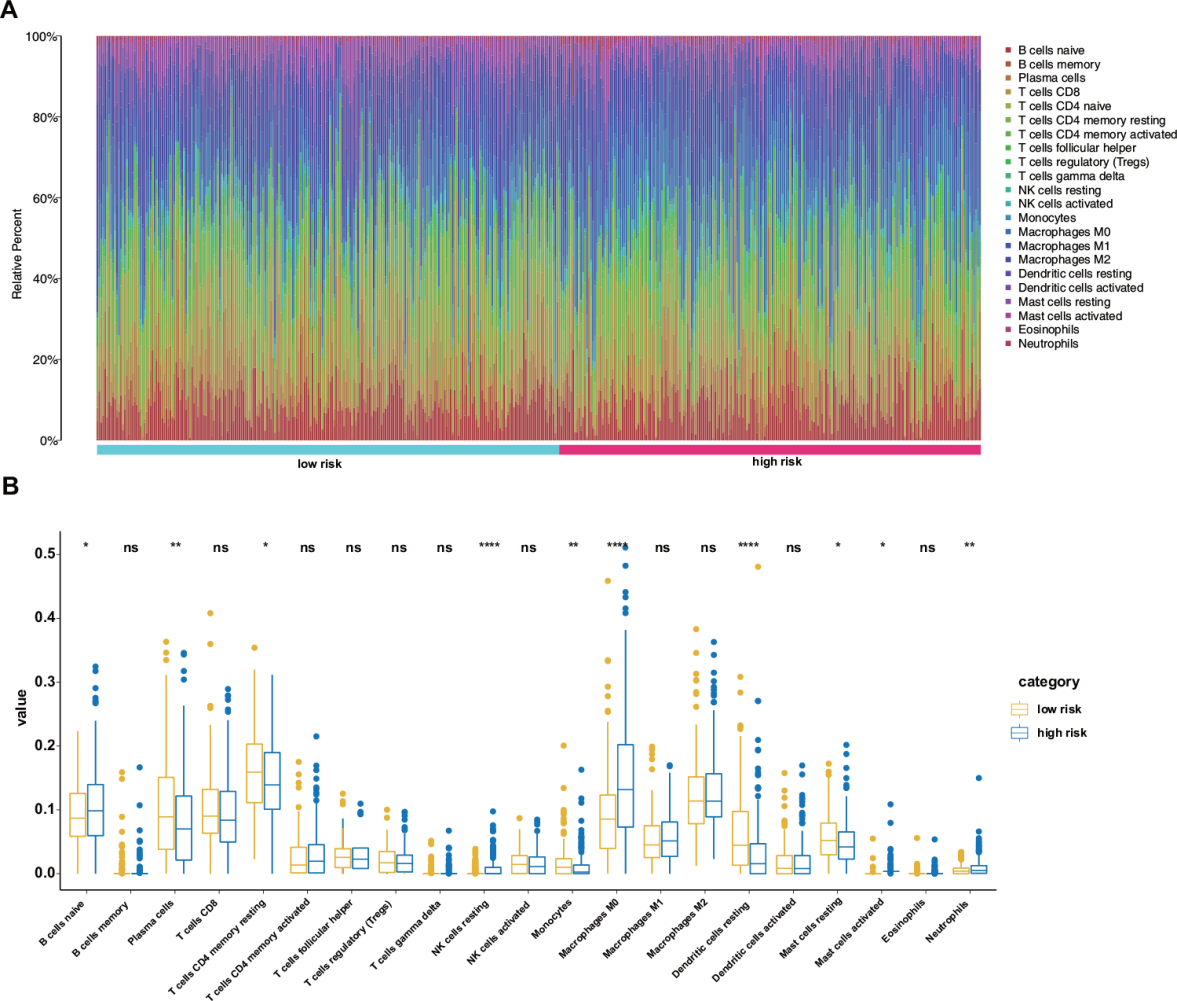
**Differences in the immune landscape between low- and high-risk patients with LUAD.** (**A**) Relative proportions of immune cell infiltration in the high- and low-risk patients. (**B**) Vioplot visualization of significantly different proportions of immune cells between low- and high-risk patients.

Using the CIBERSORT algorithm, we assessed the proportions of 22 immune cell types between the high- and low-risk patients with LUAD. Figure 8A shows the results for the 490 patients with LUAD from the training set. Regarding specific differences, the high-risk group had significantly higher proportions of naïve B cells, resting natural killer (NK) cells, M0 macrophages, activated mast cells, and neutrophils but significantly lower proportions of plasma cells, resting memory CD4 T cells, monocytes, resting dendritic cells, and resting mast cells than those in the low-risk group (Figure 8B).

### Construction and validation of a nomogram

**Figure 9:**
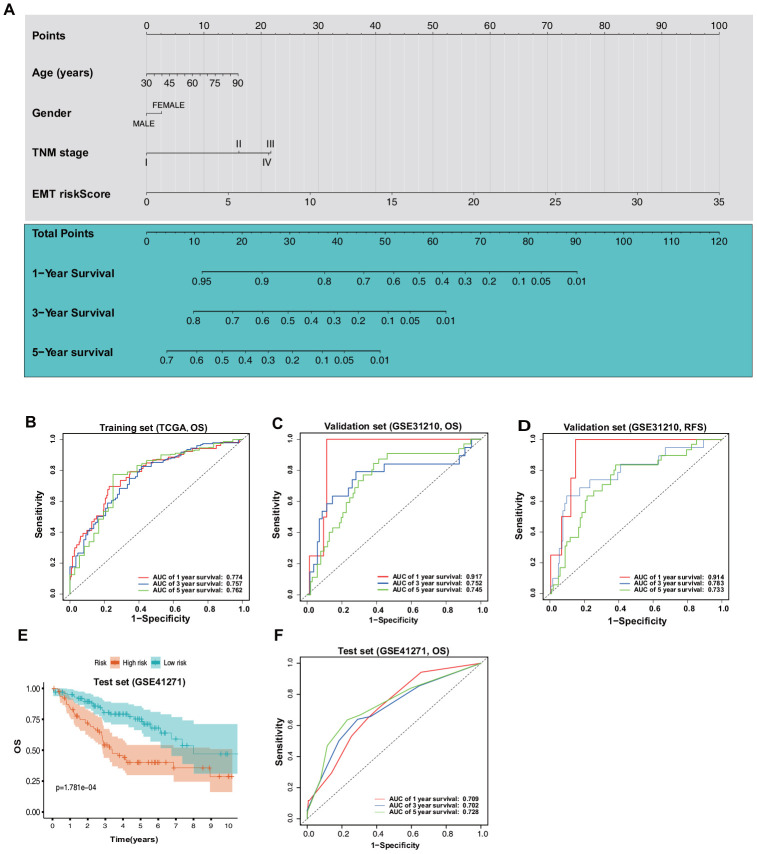
**Construction and validation of a nomogram based on the ERG signature.** (**A**) Nomogram based on the ERG signature and clinical information of patients with LUAD. (**B**) ROC curves of the nomogram for the prediction of OS in the training set. (**C**) ROC curves of the nomogram for the prediction of OS in the validation set (GSE31210). (**D**) ROC curves of the nomogram for the prediction of RFS in the validation set. (**E**) OS of the patients in the low- and high-risk scores based on the nomogram in the test set (GSE41271). (**F**) ROC curves of the nomogram for the prediction of OS in the test set.

As this novel ERG signature showed good predictive value for the LUAD prognosis, a more convenient and sensitive nomogram model, which included the ERG signature and pathological stage, age, and sex was developed based on the training set (Figure 9A). The AUC values for the 1-, 3-, and 5-year OS predictions using the nomogram were 0.774, 0.757, and 0.762, respectively (Figure 9B). In the validation set, the AUC values for 1-, 3-, and 5-year OS using the nomogram were 0.917, 0.752, and 0.745, respectively (Figure 9C), and those for 1-, 3-, and 5-year RFS were 0.914, 0.783, and 0.733, respectively (Figure 9D). To further test the nomogram, we performed survival analysis and ROC analysis by using another independent dataset, GSE41271 (test set). In the test group, survival analysis indicated that the high-risk patients had a significantly worse OS than the low-risk patients (Figure 9E). The AUC values for 1-, 3-, and 5-year OS using the nomogram were 0.709, 0.702, and 0.728, respectively (Figure 9F).

**Figure 10:**
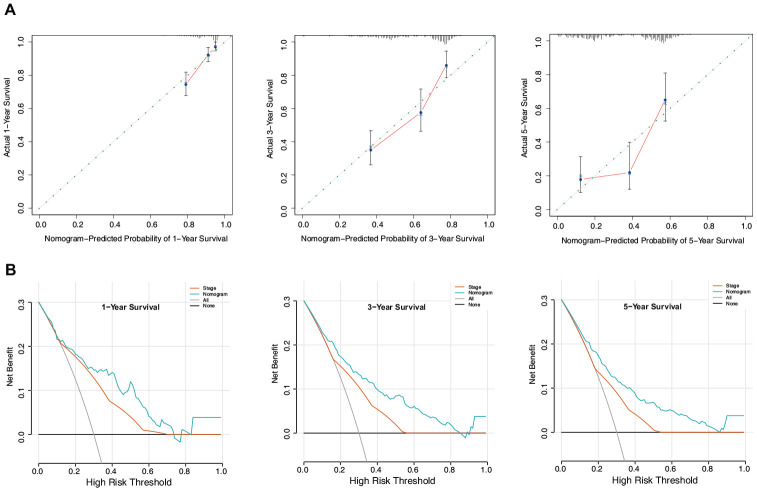
**Evaluation of the nomogram in the training set.** (**A**) Calibration plot of the nomogram for the prediction of OS. (**B**) DCA of the nomogram for the prediction of OS.

Calibration plots based on the training set showed that the nomogram could accurately predict 1-, 3-, and 5-year OS (Figure 10A). In addition, decision curve analysis (DCA) was performed for the nomogram and TNM stage and indicated marked clinical usefulness of this model (Figure 10B).

## DISCUSSION

LUAD, which is typically characterized by high recurrence and fatality and low recovery rates, is one of the deadliest malignant cancers in humans [16]. Many methods and tools have been developed to predict the survival of patients with LUAD, such as the American Joint Committee on Cancer staging system. However, TNM staging does not consider individual differences in the expression of tumor-related genes, including ERGs [17]. EMT, a process whereby epithelial cells gradually acquire a mesenchymal identity, is widely recognized to be involved in LUAD metastasis [18]. Most of the previous studies have focused on the functions and mechanisms of some ERGs [19–21]. However, ERG-based prognostic models have not been previously explored in LUAD. Herein, we systematically explored the data for patients with LUAD available in TCGA and GEO databases and established a novel ERG-based prognostic model.

In the present study, a total of 54 overlapping survival-related ERGs were identified in TCGA and GEO datasets using univariate Cox regression analysis and were then subjected to LASSO regression analysis with tenfold cross-validation. Finally, we utilized multivariate Cox analysis to select a novel, nine-ERG signature, including *ADM*, *CDH2*, *CTSL*, *FSCN1*, *FUT4*, *ITGB1*, *LGR4*, *CCR2*, and *HGF*. The patients with LUAD were categorized into high- and low-risk groups using the median risk score. The Kaplan–Meier analysis suggested that the high-risk group had a remarkably worse prognosis than that of the low-risk group. Furthermore, the estimation accuracy of the ERG signature was verified using the GSE31210 dataset, and the data indicated good reproducibility. Multivariate analyses confirmed the risk score as an independent prognostic index for LUAD.

Previous studies have shown that the process of EMT is accompanied by the release of soluble factors, which create an inflammatory milieu promoting the recruitment of immune cells to the site of tumorigenesis [22]. It is generally agreed that immune cell infiltration into the tumor microenvironment promotes tumor growth [23]. However, the modulation of immune cells by ERGs is relatively underexplored in LUAD. In this study, we assessed the immune microenvironment in the high- and low-risk groups and found that the former had distinctly higher proportions of naïve B cells, resting NK cells, M0 macrophages, activated mast cells, and neutrophils but significantly lower proportions of plasma cells, resting memory CD4 T cells, monocytes, resting dendritic cells, and resting mast cells than those in the low-risk group. Therefore, targeting ERGs may alter the tumor microenvironment and immune responses. However, as the critical roles of these immune cells are complex, further in-depth research is required.

A nomogram is an easy-to-use tool for predicting prognosis and disease incidence [24, 25]. Nomograms are currently widely used for predicting the survival of patients with malignant tumors [26, 27]. In the present study, a nomogram was established that integrated the ERG signature with the sex, age, and TNM staging and accurately predicted OS. Subsequently, ROC curve analysis, calibration plots, and DCA analysis demonstrated satisfactory predictive performance of the integrated nomogram. We also performed GSEA and GSVA to identify the most critical biological processes and signaling cascades in the high- and low-risk groups, based on our ERG signature. These analyses revealed that the chromosomal region, cell cycle, and E2F targets might play essential roles in the distinct EMT-associated risk.

In this study, nine ERGs were selected and included in the prognostic signature. ADM, a multifunctional peptide, is highly expressed in several tumors, including those of the brain, breast, colon, prostate, and lung, and plays important roles in tumor angiogenesis, cell growth, and survival [28, 29]. A previous study has investigated the prognostic role of ADM in lung cancer; however, it was found that ADM expression was not correlated with the survival of patients with lung cancer [30]. Our results were not consistent with the previous observation, likely because the previous study included squamous cell carcinoma and small-cell carcinoma. *CDH2*, which encodes the N-cadherin protein, is also a marker of EMT. *CDH2* is involved in the EMT process and promotes the growth and migration of cancer cells in LUAD [31]. It has been reported that *CDH2* has a prognostic significance for LUAD [32], which is consistent with our data. CTSL is a key effector that induces EMT in various tumors, taking part in the regulation of invasion and metastasis of cancer cells and also modulating transcription of ERGs [33, 34]. However, the critical role of *CTSL* in LUAD is unclear and needs to be studied in the future. FSCN1, an actin-bundling protein, is highly upregulated in aggressive tumors [35]. Consistent with our results, *FSCN1* expression was shown to be associated with worse survival of patients with NSCLC [36]. *FUT4*, which encodes a key glycosyltransferase, is abnormally upregulated in different types of tumors [37] and has been related to the progression and poor prognosis of LUAD [38]. *ITGB1*, a member of the integrin family, is aberrantly overexpressed or downregulated in different solid cancers [39]. It was found that low *ITGB1* levels were significantly linked to a better prognosis of NSCLC [39]. *LGR4*, also known as *GPR48*, is involved in tumorigenesis via regulating Wnt/β-catenin signaling [40]. However, few studies have focused on the prognostic value of LGR4. Further studies are needed to elucidate the critical roles of the nine ERGs identified in this study in LUAD, which may provide insights for targeted treatment of this disease.

There are some limitations to this study. First, considering the great heterogeneity of LUAD, some important clinical variables were not available from the public datasets at the time of the prognostic model construction. Thus, future studies should include more clinical variables. Second, the potential mechanisms underlying the prognostic capacity of the ERGs in LUAD remain to be explored. Third, the model has exclusively been based on the bioinformatics analysis and has not been tested in the clinic.

In conclusion, this is the first study to identify nine relevant genes and validate a novel ERG signature for the prediction of the prognosis of patients with LUAD. By integrating the ERG signature with the age, sex, and TNM staging, we constructed a predictive nomogram that could effectively estimate the outcomes of patients with LUAD via appropriate risk-score stratification. These findings could produce an effective formula for the risk score to monitor EMT and predict the prognosis of patients with LUAD.

## MATERIALS AND METHODS

### Data acquisition

We extracted 1,184 ERGs from the EMT gene database (dbEMT 2.0; http://dbemt.bioinfo-minzhao.org/download.cgi) [41] and downloaded the mRNA sequencing data, as well as clinical information, for patients with LUAD from two public databases, TCGA and GEO. After excluding samples with incomplete survival data (or follow-up times of less than 1 day), records of 490 patients with LUAD were obtained from TCGA (https://portal.gdc.cancer.gov) as a training set. Similarly, records of 226 patients with LUAD were obtained from GSE31210 (https://www.ncbi.nlm.nih.gov/geo/) as a validation set. Moreover, 179 patients with LUAD were obtained from GSE41271 as a test set. Moreover, protein expression of ERGs in LUAD and normal lung tissues was evaluated using the HPA database (https://www.proteinatlas.org/). Mutation data were obtained from the cBioPortal for Cancer Genomics (https://www.cbioportal.org/).

### Construction and validation of the ERG signature

We first identified 54 overlapping survival-related ERGs in TCGA and GSE31210 datasets, with a *P*-value of < 0.05 using univariate Cox analysis, followed by LASSO regression analysis to screen these genes. Finally, multivariate Cox regression analysis was conducted based on the ERGs selected by LASSO regression to construct a multigene signature for predicting the survival of patients with LUAD using a linear combination of regression coefficients (β_i_) derived from the LASSO Cox regression model. The risk score was calculated for each patient based on β_i_ combined with the corresponding expression data (Exp_i_) of the identified ERGs as follows:

$$\text{Risk}\;\text{Score}=\sum_{i=0}^{N}\left(\beta_{i}\times Exp_{i}\right)$$

Subsequently, the patients with LUAD were classified into low- and high-risk groups in either cohort based on the median risk score.

### Functional enrichment analysis

The GSEA software (v4.0.3; http://software.broadinstitute.org/gsea/index.jsp) was used to assess the related pathways and the molecular mechanisms by comparing the high- and low-risk groups from the training set using the KEGG gene set (c2.cp.kegg.v7.1.symbols) and GO gene set (c5.all.v7.1.symbols). For each analysis, 1,000 gene-set permutations were performed. The top five terms in each analysis were employed in multiple GSEA gene sets to demonstrate the range of biological functions and signaling pathways involved in the ERG signature in LUAD. Additionally, GSVA was performed using the GSVA package to further identify activated pathways that were determined based on the gene sets.

### Estimation of immune cell-type fractions

CIBERSORT (https://cibersort.stanford.edu/), a leukocyte gene signature matrix consisting of 547 genes, was used to estimate the putative proportions of 24 types of immune cells between the ERG signature-based low- and high-risk groups.

### Construction and validation of the nomogram

A nomogram was constructed using the “rms,” “Hmisc,” “lattice,” “Formula,” and “foreign” R packages, and the corresponding calibration map was built to evaluate the prognostic performance of the nomogram. To validate the constructed novel nomogram, we performed DCA to quantify its clinical applicability by analyzing the clinical outcomes of the nomogram-based decisions.

### Statistical analysis

The mRNA expression profiles are shown as raw data, and each mRNA expression level was log_2_-normalized for further analysis. Kaplan–Meier analysis with a log-rank test was performed for comparison between the low- and high-risk groups. ROC survival analysis was conducted to evaluate the predictive accuracy of the nomogram in patients with LUAD in terms of the ERG signature. The R software (version 3.6.2; http://www.Rproject.org) was used to conduct all statistical analyses. A *P*-value of < 0.05 was considered to indicate statistical significance.

## Supplementary Material

Supplementary Table 1

Supplementary Table 2
