# Supplementary material for: Systematic construction and validation of an epithelial–mesenchymal transition risk model to predict prognosis of lung adenocarcinoma
Source: Aging (Albany NY). 2020 Dec 3;13(1):794–812. doi: 10.18632/aging.202186 (PMC7835007; doi:10.18632/aging.202186)
Supplement: Supplementary Table 1 [file aging-13-202186-s001.docx]

| **Supplementary Table 1. Survival related ERGs in TCGA.** | | | | |
| --- | --- | --- | --- | --- |
| **Gene** | **HR** | **HR.95L** | **HR.95H** | **pvalue** |
| ACTL6A | 1.021093139 | 1.006775979 | 1.0356139 | 0.003763808 |
| ACTN4 | 1.003676999 | 1.000577881 | 1.006785716 | 0.020013091 |
| ADAM12 | 1.054172832 | 1.015086293 | 1.094764422 | 0.006205627 |
| ADIPOQ | 1.686758605 | 1.205559543 | 2.360028261 | 0.002281655 |
| ADM | 1.020640183 | 1.010848229 | 1.03052699 | 3.27221E-05 |
| AGER | 0.995532335 | 0.991995371 | 0.99908191 | 0.013671861 |
| AKTIP | 0.89457833 | 0.821191804 | 0.974523106 | 0.010744778 |
| ANGPTL4 | 1.007242662 | 1.003539571 | 1.010959418 | 0.000122958 |
| ANXA2 | 1.003513336 | 1.001414122 | 1.00561695 | 0.001028549 |
| AOC4P | 0.083281668 | 0.010949754 | 0.633423909 | 0.016347546 |
| ARRB1 | 0.960466057 | 0.932414262 | 0.989361794 | 0.007649608 |
| AURKA | 1.020769676 | 1.005821013 | 1.035940508 | 0.006312994 |
| AXL | 1.019272755 | 1.003872282 | 1.034909488 | 0.013990529 |
| BCL2L1 | 1.007786871 | 1.001506109 | 1.014107021 | 0.015024627 |
| BDNF | 1.226073575 | 1.051389619 | 1.429780535 | 0.009350163 |
| BIRC2 | 1.029866633 | 1.002599994 | 1.057874813 | 0.0315844 |
| BIRC5 | 1.019512099 | 1.005940695 | 1.033266598 | 0.004709706 |
| BMP7 | 1.032930584 | 1.011709697 | 1.054596585 | 0.002219661 |
| CAMK1D | 0.968394413 | 0.940340507 | 0.997285274 | 0.032257552 |
| CAPNS1 | 1.002771987 | 1.000229776 | 1.00532066 | 0.032568758 |
| CAPZA1 | 1.011089682 | 1.00166271 | 1.020605373 | 0.021023 |
| CCNA2 | 1.029304454 | 1.012915839 | 1.045958232 | 0.000420165 |
| CCR2 | 0.823779838 | 0.736789939 | 0.921040293 | 0.000662873 |
| CCR6 | 0.108240027 | 0.015339621 | 0.763767493 | 0.025728161 |
| CDH2 | 1.026601051 | 1.003660853 | 1.050065582 | 0.022793441 |
| CDKN1A | 1.006672731 | 1.000845162 | 1.012534233 | 0.024757792 |
| CDX2 | 1.15060455 | 1.044845668 | 1.267068306 | 0.004348279 |
| CEBPB | 1.005084174 | 1.000747472 | 1.00943967 | 0.021525369 |
| CFTR | 0.945416772 | 0.895530663 | 0.998081819 | 0.042419271 |
| CIP2A | 1.069875385 | 1.006827967 | 1.136870822 | 0.029290834 |
| CIRBP | 0.982023541 | 0.970865221 | 0.993310105 | 0.001863224 |
| CRKL | 1.011257675 | 1.002395874 | 1.02019782 | 0.012672759 |
| CRP | 1.051419351 | 1.008953196 | 1.095672877 | 0.017139334 |
| CTHRC1 | 1.003967796 | 1.000380782 | 1.007567672 | 0.030125406 |
| CTSL | 1.001785945 | 1.000690047 | 1.002883044 | 0.001397425 |
| CTSZ | 0.997849429 | 0.996059134 | 0.999642943 | 0.018786582 |
| CUL4A | 1.002066268 | 1.000177374 | 1.003958729 | 0.032016747 |
| CXCL5 | 1.009838688 | 1.005941569 | 1.013750904 | 6.94849E-07 |
| CYB5R1 | 0.977019615 | 0.957947632 | 0.996471306 | 0.020810015 |
| DKK1 | 1.008279356 | 1.004440473 | 1.012132911 | 2.27093E-05 |
| ECT2 | 1.033453054 | 1.018969569 | 1.048142405 | 4.88724E-06 |
| EFNB2 | 1.024100132 | 1.009243074 | 1.039175901 | 0.001403473 |
| EGFL7 | 0.987533998 | 0.976794691 | 0.998391378 | 0.024541988 |
| EGFR | 1.005200228 | 1.000393919 | 1.010029628 | 0.033920342 |
| EIF2AK3 | 0.910410183 | 0.848577658 | 0.976748202 | 0.008907974 |
| EIF2S1 | 1.045146257 | 1.016610538 | 1.074482957 | 0.001769937 |
| EIF4G1 | 1.010021528 | 1.003852962 | 1.016228 | 0.001421245 |
| EIF5A | 1.002640755 | 1.000594767 | 1.004690926 | 0.011390807 |
| ELF5 | 0.933860635 | 0.883132942 | 0.987502157 | 0.016336606 |
| ENO1 | 1.001324035 | 1.000634173 | 1.002014373 | 0.000167954 |
| EPHA2 | 1.013476983 | 1.007170855 | 1.019822597 | 2.6264E-05 |
| ERF | 1.021251757 | 1.003225739 | 1.039601668 | 0.020645272 |
| ERG | 0.876190853 | 0.774780648 | 0.99087453 | 0.035201041 |
| ERN1 | 0.928823933 | 0.864657592 | 0.997752067 | 0.043219749 |
| ERRFI1 | 1.001958478 | 1.000207525 | 1.003712496 | 0.028344213 |
| ESRP1 | 1.013903634 | 1.000550521 | 1.027434955 | 0.041218069 |
| FBLN5 | 1.010344654 | 1.003266447 | 1.017472799 | 0.004116225 |
| FBP1 | 0.994244869 | 0.990059197 | 0.998448238 | 0.007330574 |
| FBXO45 | 1.085916526 | 1.027898808 | 1.147208941 | 0.003258982 |
| FERMT1 | 1.034237123 | 1.015704912 | 1.053107468 | 0.000263148 |
| FGF2 | 1.278966384 | 1.120300122 | 1.460104288 | 0.00027172 |
| FHL1 | 0.965757427 | 0.938022693 | 0.9943122 | 0.019096693 |
| FKBP5 | 1.017475093 | 1.003184651 | 1.031969104 | 0.016370508 |
| FOSL1 | 1.011141242 | 1.003204028 | 1.019141255 | 0.005859489 |
| FOXA2 | 0.985727023 | 0.972124951 | 0.999519415 | 0.042583853 |
| FOXC2 | 1.14761111 | 1.030295316 | 1.278285206 | 0.012335001 |
| FOXM1 | 1.029594035 | 1.013354082 | 1.04609425 | 0.000323996 |
| FSCN1 | 1.008809905 | 1.005513218 | 1.012117402 | 1.50363E-07 |
| FST | 1.013566943 | 1.00249661 | 1.024759522 | 0.016173771 |
| FURIN | 1.000944271 | 1.000340468 | 1.001548439 | 0.00217193 |
| FUT4 | 1.169844156 | 1.088404173 | 1.257377895 | 2.03571E-05 |
| G3BP1 | 1.04128254 | 1.006702382 | 1.077050523 | 0.018893427 |
| GAPDH | 1.000648303 | 1.00037738 | 1.0009193 | 2.71922E-06 |
| GATA1 | 0.130626569 | 0.027999722 | 0.609409645 | 0.009591223 |
| GDF15 | 0.997523517 | 0.995055952 | 0.9999972 | 0.049741538 |
| GIPC2 | 1.130034649 | 1.037961416 | 1.230275315 | 0.004814467 |
| GLI1 | 1.10214025 | 1.011113206 | 1.201362146 | 0.027018554 |
| GLI2 | 1.388190939 | 1.191898857 | 1.616810078 | 2.47763E-05 |
| GLS2 | 0.483135741 | 0.240358508 | 0.971133271 | 0.041132261 |
| GPI | 1.007693203 | 1.00233702 | 1.013078008 | 0.004826013 |
| GSK3A | 1.038722593 | 1.015616704 | 1.062354154 | 0.000932711 |
| H2AFX | 1.010576626 | 1.003656283 | 1.017544686 | 0.002691374 |
| H2AFZ | 1.004433946 | 1.00131346 | 1.007564157 | 0.005323629 |
| HAS2 | 1.046136592 | 1.010102636 | 1.083456007 | 0.011668056 |
| HAS2-AS1 | 1.485274559 | 1.129518096 | 1.953081161 | 0.00462921 |
| HDAC2 | 1.060355717 | 1.017351293 | 1.10517798 | 0.005531598 |
| HDAC4 | 1.044241135 | 1.009514918 | 1.080161895 | 0.012115395 |
| HDAC5 | 0.946726998 | 0.900791138 | 0.995005357 | 0.030984052 |
| HGF | 0.824927865 | 0.709525679 | 0.959099864 | 0.012311525 |
| HK2 | 1.013636594 | 1.001553857 | 1.025865097 | 0.0268472 |
| HMGA2 | 1.040533274 | 1.012404554 | 1.069443524 | 0.004487955 |
| HNF4A | 1.069254937 | 1.012794461 | 1.128862928 | 0.015551043 |
| HNRNPAB | 1.00716813 | 1.001323202 | 1.013047176 | 0.016161058 |
| HOXA10 | 1.117875828 | 1.033776794 | 1.208816423 | 0.005231487 |
| HOXA13 | 1.145374151 | 1.074002967 | 1.221488195 | 3.55232E-05 |
| HOXD9 | 1.133746191 | 1.040231027 | 1.235668223 | 0.00426328 |
| HSP90AA1 | 1.001679462 | 1.000348418 | 1.003012277 | 0.013381905 |
| HSPA4 | 1.034010762 | 1.016588412 | 1.051731698 | 0.000114514 |
| IGF1R | 1.03053825 | 1.004974806 | 1.056751948 | 0.01891636 |
| IGFBP3 | 1.00212827 | 1.000684442 | 1.003574181 | 0.003851597 |
| IL11 | 1.143623987 | 1.05283727 | 1.242239291 | 0.001472594 |
| IRF8 | 0.95300874 | 0.913617148 | 0.994098743 | 0.025432026 |
| ITGA2 | 1.011091512 | 1.002200827 | 1.020061068 | 0.014372181 |
| ITGA5 | 1.008700142 | 1.004412282 | 1.013006307 | 6.73226E-05 |
| ITGA6 | 1.007938277 | 1.002867861 | 1.013034329 | 0.002119719 |
| ITGB1 | 1.006101117 | 1.003559509 | 1.008649162 | 2.43805E-06 |
| ITGB4 | 1.005786033 | 1.002156188 | 1.009429025 | 0.001762526 |
| JAG1 | 1.032036431 | 1.014487671 | 1.049888752 | 0.00031364 |
| KAT8 | 0.957019278 | 0.91631059 | 0.999536521 | 0.047606196 |
| KDM1A | 1.017822755 | 1.002148104 | 1.033742574 | 0.02568448 |
| KHDRBS1 | 1.018256866 | 1.006372767 | 1.030281303 | 0.002523274 |
| KLF4 | 1.016655876 | 1.001525265 | 1.032015075 | 0.030836076 |
| KLF5 | 1.007778267 | 1.001437633 | 1.014159047 | 0.016124785 |
| KLK6 | 1.009313876 | 1.001366062 | 1.017324772 | 0.021538664 |
| KMT5A | 1.064868195 | 1.005155864 | 1.128127799 | 0.032791743 |
| KRAS | 1.012773035 | 1.005184895 | 1.020418458 | 0.000940608 |
| KRT17 | 1.001211672 | 1.00026958 | 1.002154651 | 0.011697262 |
| KRT18 | 1.000862061 | 1.00047649 | 1.001247781 | 1.16981E-05 |
| KRT19 | 1.000753149 | 1.000251216 | 1.001255333 | 0.003268463 |
| KRT7 | 1.001730937 | 1.001091424 | 1.002370859 | 1.1094E-07 |
| KRT8 | 1.001345441 | 1.000761844 | 1.001929379 | 6.17592E-06 |
| L1CAM | 1.021705409 | 1.002060877 | 1.041735055 | 0.030173639 |
| LAMC2 | 1.002393678 | 1.001274911 | 1.003513695 | 2.71494E-05 |
| LGR4 | 1.026621803 | 1.01101462 | 1.042469916 | 0.000775239 |
| LGR5 | 1.021353093 | 1.000403305 | 1.042741597 | 0.045706176 |
| LHX2 | 1.175094166 | 1.027238498 | 1.344231454 | 0.018690096 |
| LMNB1 | 1.017586416 | 1.006106995 | 1.029196814 | 0.002597142 |
| LOX | 1.02239165 | 1.007053429 | 1.037963484 | 0.00408769 |
| LOXL2 | 1.016287081 | 1.010664433 | 1.02194101 | 1.14614E-08 |
| LRIG1 | 0.964798894 | 0.94121082 | 0.98897812 | 0.004546152 |
| LTBP1 | 1.015361961 | 1.000369289 | 1.030579331 | 0.044578476 |
| LYPD3 | 1.011937501 | 1.00549804 | 1.018418202 | 0.000269114 |
| MAP2K1 | 1.025271657 | 1.003720943 | 1.047285083 | 0.021299858 |
| MAP3K3 | 0.93067984 | 0.874630068 | 0.990321504 | 0.023399503 |
| MAP3K7 | 1.087963818 | 1.009400917 | 1.172641365 | 0.027478706 |
| MAP4K4 | 1.035607856 | 1.017369744 | 1.054172917 | 0.000113595 |
| MARVELD1 | 1.02930033 | 1.015500069 | 1.043288132 | 2.74861E-05 |
| MCL1 | 1.003036243 | 1.000182672 | 1.005897954 | 0.037012855 |
| MEG3 | 1.020856331 | 1.009326456 | 1.032517916 | 0.000368294 |
