# Supplementary material for: Systematic construction and validation of an epithelial–mesenchymal transition risk model to predict prognosis of lung adenocarcinoma
Source: Aging (Albany NY). 2020 Dec 3;13(1):794–812. doi: 10.18632/aging.202186 (PMC7835007; doi:10.18632/aging.202186)
Supplement: Supplementary Table 2 [file aging-13-202186-s002.docx]

| **Supplementary Table 2. Survival related ERGs in GSE31210.** | | | | |
| --- | --- | --- | --- | --- |
| **Gene** | **HR** | **HR.95L** | **HR.95H** | **pvalue** |
| ABCB1 | 0.572363948 | 0.364788623 | 0.898055662 | 0.015190564 |
| ACTN4 | 3.120304831 | 1.333971568 | 7.298732949 | 0.008675181 |
| ADAM10 | 12.78355763 | 3.992022836 | 40.93647565 | 1.78E-05 |
| ADAM12 | 1.309917254 | 1.040077863 | 1.649764188 | 0.021798875 |
| ADAM17 | 3.080594993 | 1.066731574 | 8.896395062 | 0.037585125 |
| ADM | 1.845427748 | 1.374286248 | 2.478088955 | 4.62E-05 |
| AFAP1L2 | 1.90297279 | 1.273589531 | 2.843385054 | 0.001687758 |
| AGR2 | 1.328324589 | 1.010424891 | 1.746241831 | 0.041924226 |
| AGTR1 | 0.596250106 | 0.451849364 | 0.786798029 | 0.000257491 |
| ANXA2 | 42.2174006 | 7.888511045 | 225.9373034 | 1.22E-05 |
| ANXA5 | 3.70367879 | 1.062043473 | 12.91588991 | 0.039935698 |
| APBB1 | 0.640527134 | 0.423852408 | 0.967966682 | 0.034471992 |
| AQP3 | 0.68782573 | 0.516825985 | 0.915403344 | 0.010285874 |
| AQP5 | 0.855248561 | 0.732584714 | 0.998451221 | 0.047749952 |
| ARHGEF2 | 0.411936346 | 0.206356967 | 0.822320448 | 0.011915784 |
| ARRB1 | 0.302244959 | 0.167388341 | 0.545748975 | 7.23E-05 |
| ATXN1 | 0.317325418 | 0.101038429 | 0.996605165 | 0.049323113 |
| AURKA | 1.393063734 | 1.035834067 | 1.873491739 | 0.02831804 |
| BAG3 | 2.617408635 | 1.238777909 | 5.530311699 | 0.011702644 |
| BHLHE40 | 2.195182813 | 1.274266964 | 3.781646797 | 0.004606055 |
| BIRC5 | 1.504721593 | 1.169781938 | 1.935563372 | 0.001469467 |
| BRAF | 0.328410742 | 0.14161461 | 0.761599495 | 0.009472137 |
| BRCA1 | 1.892155313 | 1.248066574 | 2.868638424 | 0.002667203 |
| BRMS1 | 5.394580957 | 1.897512803 | 15.33665736 | 0.0015695 |
| BSG | 2.089960303 | 1.24394804 | 3.511347681 | 0.005360189 |
| BTBD7 | 0.261582352 | 0.098794702 | 0.692601176 | 0.006948522 |
| BTRC | 0.1841222 | 0.039720174 | 0.853495373 | 0.030587172 |
| CA9 | 1.463459893 | 1.185741368 | 1.806224288 | 0.000390015 |
| CAMK1D | 0.66211563 | 0.456377464 | 0.960602004 | 0.029880377 |
| CAPNS1 | 2.115584653 | 1.193839459 | 3.74899522 | 0.010261569 |
| CAV1 | 0.629368668 | 0.441887173 | 0.896393797 | 0.010284556 |
| CCL20 | 1.320908545 | 1.132736584 | 1.540339925 | 0.000385985 |
| CCNA2 | 1.630434958 | 1.128158493 | 2.356333945 | 0.009274851 |
| CCR2 | 0.662336888 | 0.441146804 | 0.994431219 | 0.046933914 |
| CCR6 | 0.65288835 | 0.433869538 | 0.982468598 | 0.040875448 |
| CD14 | 1.697094446 | 1.18257036 | 2.435482622 | 0.004106976 |
| CD63 | 7.062905192 | 1.362805162 | 36.60437394 | 0.01987458 |
| CDH2 | 1.307417168 | 1.003631968 | 1.703153852 | 0.046940611 |
| CDK5 | 2.31594567 | 1.364256115 | 3.931523038 | 0.001868793 |
| CDKL2 | 0.798475062 | 0.64743848 | 0.984745954 | 0.035409124 |
| CDKN1B | 0.35418991 | 0.163243945 | 0.76848481 | 0.008632181 |
| CIRBP | 0.249151465 | 0.137310481 | 0.452088231 | 4.84E-06 |
| CKS2 | 1.649322086 | 1.061847114 | 2.561822044 | 0.025943197 |
| CNTN1 | 0.690479296 | 0.520060683 | 0.916742361 | 0.010435081 |
| CREB1 | 0.072041279 | 0.013300779 | 0.390198643 | 0.002274939 |
| CREBBP | 0.213792772 | 0.073659841 | 0.620519246 | 0.004543705 |
| CRIM1 | 0.392170203 | 0.253993748 | 0.605516746 | 2.41E-05 |
| CRK | 0.238515267 | 0.072879644 | 0.780595646 | 0.017815265 |
| CSNK2B | 2.484071549 | 1.054054606 | 5.854166783 | 0.037495648 |
| CTBP1 | 0.169231465 | 0.033264715 | 0.860950977 | 0.032326753 |
| CTHRC1 | 2.061380139 | 1.352226002 | 3.142439262 | 0.000771864 |
| CTSL | 1.980300822 | 1.11100949 | 3.529755041 | 0.020507127 |
| CTSZ | 1.901429033 | 1.018290575 | 3.550491829 | 0.043711042 |
| CUX1 | 0.318577955 | 0.119249038 | 0.851092093 | 0.022515491 |
| CXCL8 | 1.478916467 | 1.155288617 | 1.893201305 | 0.001899051 |
| CYP4Z1 | 0.760732833 | 0.605924891 | 0.955092705 | 0.018484801 |
| CYP7B1 | 1.716408596 | 1.212456489 | 2.429826138 | 0.002317004 |
| DAPK1 | 0.577642816 | 0.37230428 | 0.896232572 | 0.014332748 |
| DDX5 | 0.490138687 | 0.282574471 | 0.850168565 | 0.011160868 |
| DVL2 | 0.264232103 | 0.094535153 | 0.738546476 | 0.011152802 |
| ECT2 | 1.759077429 | 1.222213692 | 2.531761361 | 0.002365248 |
| EED | 2.703157239 | 1.024121525 | 7.134953105 | 0.044633102 |
| EGLN3 | 1.608331897 | 1.278085227 | 2.023911581 | 5.07E-05 |
| EIF4E | 0.153581665 | 0.04376326 | 0.53897557 | 0.003445605 |
| EIF4G1 | 2.908629451 | 1.014882091 | 8.336067174 | 0.046871168 |
| ENG | 0.505319606 | 0.26545053 | 0.961941587 | 0.037700423 |
| EP300 | 0.324543031 | 0.142829036 | 0.737442345 | 0.007204125 |
| EPAS1 | 0.305970252 | 0.151216151 | 0.619099183 | 0.000989801 |
| EPB41L5 | 0.415755618 | 0.252114345 | 0.68561245 | 0.000584133 |
| EPHA4 | 0.68159829 | 0.496194545 | 0.936278388 | 0.017959494 |
| EPHB2 | 2.197906236 | 1.423687117 | 3.393155537 | 0.000378957 |
| EPO | 0.605461343 | 0.400269179 | 0.915842282 | 0.01748718 |
| ERG | 0.530156814 | 0.312169009 | 0.90036563 | 0.018856163 |
| ERN1 | 0.402442956 | 0.196346041 | 0.824871906 | 0.012927724 |
| ETS1 | 0.479405709 | 0.250386318 | 0.917900925 | 0.02652371 |
| ETS2 | 0.605858309 | 0.390250945 | 0.94058527 | 0.025555386 |
| EXOC4 | 3.918402068 | 1.279040753 | 12.00421076 | 0.016811099 |
| EZH2 | 1.648720026 | 1.183203852 | 2.297387488 | 0.00313923 |
| FBXO11 | 0.059213252 | 0.015795433 | 0.221976134 | 2.76E-05 |
| FBXO45 | 1.953412434 | 1.064515704 | 3.584559743 | 0.030632233 |
| FGF1 | 1.660722236 | 1.124494846 | 2.452655389 | 0.010779888 |
| FGF9 | 0.762300302 | 0.584877039 | 0.993545159 | 0.04465707 |
| FGFR2 | 0.620494266 | 0.43178043 | 0.89168732 | 0.00989067 |
| FHL1 | 0.670558445 | 0.515435969 | 0.872365637 | 0.00290916 |
| FHL2 | 1.512179911 | 1.117467214 | 2.046313354 | 0.007370881 |
| FHOD1 | 0.392335216 | 0.183385832 | 0.839361035 | 0.015897727 |
| FLT1 | 1.812895987 | 1.075975653 | 3.054522518 | 0.025412535 |
| FOXM1 | 1.654449843 | 1.290993219 | 2.120231341 | 6.95E-05 |
| FSCN1 | 1.770160794 | 1.026206381 | 3.05344938 | 0.040076368 |
| FURIN | 1.926952313 | 1.237232198 | 3.00117086 | 0.003711903 |
| FUT4 | 1.65914492 | 1.066145649 | 2.581975425 | 0.024844046 |
| GAPDH | 5.967066266 | 2.441121757 | 14.58586804 | 8.97E-05 |
| GJB2 | 1.239039663 | 1.054461241 | 1.45592766 | 0.009206024 |
| GLS | 0.454164652 | 0.2914942 | 0.707614529 | 0.000485529 |
| GLS2 | 0.755587961 | 0.606030658 | 0.942053276 | 0.012759767 |
| GPC3 | 0.644302665 | 0.46394746 | 0.894769256 | 0.008701292 |
| GPI | 2.265551395 | 1.43321836 | 3.581256888 | 0.00046427 |
| GPR32 | 1.428361421 | 1.004030455 | 2.03202636 | 0.047442469 |
| GREM1 | 1.309537514 | 1.11437813 | 1.538874872 | 0.001055692 |
| GRN | 2.544427692 | 1.211146795 | 5.3454398 | 0.013672594 |
| H2AFX | 1.96292585 | 1.24117724 | 3.104373629 | 0.003928909 |
| HAVCR2 | 1.905635325 | 1.083946912 | 3.350206502 | 0.025091792 |
| HDAC2 | 2.610587294 | 1.203483443 | 5.662866459 | 0.015149995 |
| HDAC4 | 0.294157131 | 0.144692113 | 0.598017513 | 0.000724254 |
| HES1 | 1.747770435 | 1.129620309 | 2.70418429 | 0.01216625 |
| HGF | 0.571027351 | 0.35468958 | 0.919317211 | 0.021098926 |
| HIF1A | 6.443564187 | 1.421270928 | 29.21295203 | 0.015700161 |
| HIP1 | 0.50971133 | 0.319360362 | 0.813518743 | 0.004725402 |
| HMGB3 | 1.499473351 | 1.107452083 | 2.03026421 | 0.008791911 |
| HMOX1 | 2.475978608 | 1.751982227 | 3.499162248 | 2.79E-07 |
| HNF4A | 1.300930694 | 1.005230652 | 1.68361427 | 0.045541582 |
| HOXA13 | 1.325116427 | 1.016157685 | 1.728012858 | 0.037681568 |
| HPGD | 0.805902983 | 0.650447052 | 0.998512663 | 0.048429872 |
| HSF1 | 1.947576172 | 1.186739278 | 3.196197358 | 0.008355485 |
| HSPA5 | 2.943946248 | 1.168430028 | 7.417491251 | 0.022014607 |
| IDH2 | 2.099247273 | 1.296239276 | 3.399711146 | 0.002571529 |
| IGFBP3 | 1.63979877 | 1.133322732 | 2.372616317 | 0.008691145 |
| IGFBP7 | 0.449488532 | 0.205133281 | 0.984920338 | 0.045724492 |
| IKBKG | 6.408472338 | 2.838715456 | 14.46728929 | 7.77E-06 |
| IL1B | 1.760159469 | 1.226865588 | 2.525265512 | 0.002138981 |
| IL23A | 1.653968203 | 1.226033988 | 2.231268336 | 0.000987612 |
| IL6R | 0.47216984 | 0.32041201 | 0.695805248 | 0.000148644 |
| INPP4B | 1.605446016 | 1.094668566 | 2.354554604 | 0.015397096 |
| ISG15 | 1.35957473 | 1.046413521 | 1.766456003 | 0.021470236 |
| ITGA3 | 1.538479817 | 1.059752583 | 2.233464855 | 0.023505699 |
| ITGB1 | 4.439023406 | 1.05873767 | 18.61171974 | 0.041548815 |
| ITGB4 | 1.539043218 | 1.113300017 | 2.127597224 | 0.009065876 |
| KAT2B | 0.570580188 | 0.333646265 | 0.975769206 | 0.040407099 |
| KAT8 | 0.379095541 | 0.161387475 | 0.890486882 | 0.026003148 |
| KCNN4 | 1.471186472 | 1.151002601 | 1.880438526 | 0.002049164 |
| KDM5A | 3.733544199 | 1.111316969 | 12.54309317 | 0.033116183 |
| KDM6B | 0.338612064 | 0.132202913 | 0.867288982 | 0.024028134 |
| KIF5B | 0.240893843 | 0.067413481 | 0.860804735 | 0.028477527 |
| KL | 0.616797494 | 0.443462931 | 0.857882637 | 0.004097096 |
| KLF17 | 0.571583928 | 0.424128669 | 0.770304418 | 0.000238575 |
| KRT18 | 2.343590234 | 1.279203542 | 4.293620996 | 0.005831834 |
| KRT19 | 1.982943908 | 1.331114192 | 2.953966358 | 0.000761397 |
| KRT7 | 1.64817572 | 1.041808762 | 2.607468187 | 0.032763486 |
| KRT8 | 2.206874173 | 1.48084993 | 3.288850217 | 0.000100758 |
| L1CAM | 1.330983351 | 1.001830881 | 1.768279171 | 0.048542988 |
| LAMC2 | 1.436584101 | 1.105159249 | 1.867399545 | 0.00678599 |
| LATS1 | 0.241191869 | 0.088678153 | 0.656007324 | 0.005340012 |
| LCN2 | 1.346673422 | 1.137832844 | 1.593845103 | 0.00053653 |
| LETMD1 | 0.452155932 | 0.21958426 | 0.931054833 | 0.031254976 |
| LGALS1 | 1.84755299 | 1.205459798 | 2.8316598 | 0.004837362 |
| LGALS9 | 3.983771938 | 1.862550601 | 8.520809499 | 0.000366212 |
| LGR4 | 0.720946625 | 0.541706384 | 0.95949402 | 0.024865328 |
| LHX2 | 1.437287515 | 1.059944657 | 1.948965342 | 0.019562505 |
| LMNB1 | 1.746105105 | 1.115409924 | 2.733419324 | 0.014784113 |
| LOX | 1.56258392 | 1.069229599 | 2.283577362 | 0.021123902 |
| LOXL2 | 1.484537558 | 1.08380629 | 2.033436953 | 0.013842977 |
| LOXL3 | 2.171985249 | 1.170209912 | 4.031345035 | 0.013967625 |
| LRIG1 | 0.555773946 | 0.347891356 | 0.887876843 | 0.013990623 |
| LYN | 2.853107574 | 1.503517484 | 5.414119167 | 0.00133811 |
| LYPD3 | 1.207188852 | 1.004279707 | 1.451094665 | 0.044915157 |
| MAGEC2 | 1.329181951 | 1.019221822 | 1.733405448 | 0.0356846 |
| MALAT1 | 0.360371495 | 0.147755383 | 0.878936598 | 0.024855604 |
| MAP3K3 | 0.19611771 | 0.078722929 | 0.488576289 | 0.00046885 |
| MAPK14 | 3.421188761 | 1.581426202 | 7.401251173 | 0.001783606 |
| MAPK3 | 3.212843414 | 1.141030195 | 9.046529046 | 0.027122761 |
| MDK | 1.916364409 | 1.347773329 | 2.724829517 | 0.000292456 |
| MDM4 | 0.242484139 | 0.109476305 | 0.537089347 | 0.000479469 |
| MICA | 0.586913045 | 0.393401265 | 0.875612137 | 0.009034303 |
| MIR34A | 0.416526689 | 0.2040023 | 0.850453562 | 0.016184224 |
| MIRLET7D | 0.631939433 | 0.411740286 | 0.969901322 | 0.035747981 |
| MKL2 | 0.334452231 | 0.143542471 | 0.779269675 | 0.011153621 |
| MMD | 1.641402286 | 1.056642593 | 2.549775565 | 0.027444538 |
| MMP1 | 1.181550663 | 1.033528768 | 1.350772239 | 0.014570637 |
| MMP11 | 1.326067457 | 1.098230876 | 1.601170519 | 0.003344683 |
| MMP14 | 2.193672367 | 1.328138687 | 3.623265027 | 0.002152313 |
| MMP9 | 1.250396721 | 1.018262298 | 1.535451094 | 0.032950586 |
| MSX2 | 1.842994037 | 1.114448712 | 3.047809186 | 0.017211094 |
| MTA3 | 2.741425607 | 1.30341473 | 5.765942481 | 0.007848587 |
| MTHFD2 | 1.866712524 | 1.160219869 | 3.003409731 | 0.010098783 |
| MUC16 | 1.191038011 | 1.063740565 | 1.333569096 | 0.002434166 |
| MYBL2 | 1.358488244 | 1.129500543 | 1.633899443 | 0.001142258 |
| NANOG | 0.536546355 | 0.343095633 | 0.839072151 | 0.006351686 |
| NCOA3 | 0.339811352 | 0.13865943 | 0.832772463 | 0.018270371 |
| NDRG1 | 2.382189091 | 1.47148393 | 3.856532002 | 0.000413233 |
| NEDD4L | 0.615512367 | 0.405613631 | 0.934030429 | 0.022566775 |
| NKX3-2 | 1.356541771 | 1.064394994 | 1.7288747 | 0.013728599 |
| NME1 | 1.828670746 | 1.170236452 | 2.857573522 | 0.008043999 |
| NMI | 2.412720158 | 1.213399024 | 4.797447867 | 0.012020926 |
| NOTCH1 | 0.445112504 | 0.245445967 | 0.807204713 | 0.00769467 |
| NR1I2 | 1.301275147 | 1.0497046 | 1.613136695 | 0.016282839 |
| NR2C2 | 0.202587658 | 0.072958898 | 0.562532605 | 0.002183607 |
| NR2F2 | 0.467143678 | 0.255397133 | 0.854446615 | 0.013490284 |
| NTN4 | 0.670816053 | 0.47592986 | 0.945505241 | 0.022610444 |
| NTRK3 | 0.560460007 | 0.32691293 | 0.960853461 | 0.035277703 |
| NUMB | 0.452525472 | 0.273265715 | 0.749377955 | 0.002062753 |
| OCLN | 0.623254836 | 0.431228893 | 0.900789805 | 0.011870765 |
| PAG1 | 0.481674585 | 0.268704998 | 0.863439115 | 0.014165518 |
| PAK1 | 4.404206366 | 2.113856148 | 9.17613705 | 7.54E-05 |
| PAQR3 | 0.501051433 | 0.268219782 | 0.93599561 | 0.030202705 |
| PBXIP1 | 0.477304796 | 0.239901566 | 0.949638936 | 0.035101443 |
| PCGF2 | 0.400507967 | 0.168984941 | 0.949236249 | 0.037682338 |
| PDPN | 1.653353853 | 1.157631013 | 2.361356022 | 0.005694633 |
| PEAK1 | 0.537818879 | 0.311545174 | 0.928434045 | 0.025978713 |
| PEBP4 | 0.841197928 | 0.710934095 | 0.99532989 | 0.043957593 |
| PGF | 0.477415586 | 0.259899599 | 0.876975736 | 0.017168256 |
| PHLDA2 | 1.543701376 | 1.06800135 | 2.231283638 | 0.02088922 |
| PIK3R1 | 0.465684039 | 0.268264074 | 0.808388618 | 0.006610203 |
| PITPNM3 | 0.506850014 | 0.3349763 | 0.766910784 | 0.001300448 |
| PKP3 | 1.861141626 | 1.15006256 | 3.011878026 | 0.011430951 |
| PLAUR | 1.807830834 | 1.327213561 | 2.462491659 | 0.00017316 |
| POSTN | 1.977030842 | 1.234201734 | 3.166946574 | 0.004578552 |
| PPARGC1A | 0.753682676 | 0.569330338 | 0.997729329 | 0.048172052 |
| PPM1D | 0.313964827 | 0.116247102 | 0.847968771 | 0.022296495 |
| PRKCE | 0.324370618 | 0.130077151 | 0.808876096 | 0.01573834 |
| PRMT1 | 1.909971561 | 1.066279008 | 3.421235282 | 0.029574448 |
| PSME3 | 3.666401239 | 1.721947755 | 7.806565561 | 0.000753441 |
| PTP4A2 | 4.569950523 | 1.265035784 | 16.50897788 | 0.020410164 |
| PTPN14 | 0.546731597 | 0.307616749 | 0.971713797 | 0.039613791 |
| RAB43 | 1.751265966 | 1.146454829 | 2.675144632 | 0.009535058 |
| RAC1 | 9.524924592 | 1.994125778 | 45.49572021 | 0.004726965 |
| RAF1 | 0.222323235 | 0.05827474 | 0.84818261 | 0.027736664 |
| RGCC | 0.708040754 | 0.502980279 | 0.996702515 | 0.047827778 |
| RHOC | 2.7759371 | 1.625079826 | 4.741814317 | 0.000185962 |
| RHOG | 2.338295119 | 1.159874365 | 4.713979573 | 0.017569337 |
| RUNX2 | 1.826964079 | 1.239473069 | 2.692916717 | 0.002330458 |
| S100A2 | 1.264105524 | 1.084851277 | 1.472978657 | 0.002666317 |
| S100A8 | 1.289540318 | 1.055611801 | 1.575308488 | 0.012778005 |
| S100A9 | 1.367445449 | 1.148528162 | 1.628089861 | 0.000438599 |
| S100P | 1.137648347 | 1.011062658 | 1.280082644 | 0.032132041 |
| SEMA7A | 2.30041595 | 1.281723182 | 4.128749184 | 0.00524308 |
| SERPINB4 | 1.246692147 | 1.054122098 | 1.474441444 | 0.01000464 |
| SERPINI1 | 1.641235739 | 1.138449624 | 2.366072853 | 0.007936523 |
| SFRP2 | 1.316417337 | 1.07471126 | 1.612483901 | 0.007905026 |
| SIRT1 | 0.172866452 | 0.065259476 | 0.457907603 | 0.000413217 |
| SIX1 | 1.251616469 | 1.002267368 | 1.562999889 | 0.047709848 |
| SKP2 | 1.950031602 | 1.241025422 | 3.064097789 | 0.003773428 |
| SLC2A1 | 1.701546597 | 1.290337615 | 2.243800992 | 0.000165903 |
| SLC30A7 | 3.664308129 | 1.240933279 | 10.82020629 | 0.018737872 |
| SLC9A3R1 | 1.667611852 | 1.116194466 | 2.491437982 | 0.012538385 |
| SLIT3 | 0.670058428 | 0.46357716 | 0.968508233 | 0.033154678 |
| SMAD3 | 2.33251135 | 1.150092003 | 4.730586063 | 0.018895779 |
| SMAD4 | 0.467296373 | 0.248624282 | 0.878296757 | 0.018125974 |
| SMAD9 | 0.522436046 | 0.342820649 | 0.796158057 | 0.002523777 |
| SNW1 | 0.256242864 | 0.074077102 | 0.886379234 | 0.031519942 |
| SON | 0.15296675 | 0.054659601 | 0.428082641 | 0.000349081 |
| SOX3 | 1.878953295 | 1.212014614 | 2.912890195 | 0.004809086 |
| SPHK1 | 1.630916117 | 1.161196964 | 2.290642726 | 0.004768412 |
| SPOCK1 | 1.21898643 | 1.002714062 | 1.481905933 | 0.046904316 |
| SPOP | 0.130582703 | 0.037111029 | 0.459481793 | 0.001516733 |
| SPP1 | 1.759898058 | 1.299829375 | 2.382805957 | 0.000256084 |
| SPRY1 | 0.498659411 | 0.300458843 | 0.827604892 | 0.0071023 |
| SPRY4-IT1 | 0.596034216 | 0.489453762 | 0.725822978 | 2.63E-07 |
| SRC | 3.235682832 | 1.423924993 | 7.35266495 | 0.005049564 |
| ST14 | 1.724321466 | 1.172740734 | 2.53532979 | 0.005603545 |
| STAT1 | 1.776198013 | 1.042558659 | 3.026092925 | 0.034576662 |
| STAT5B | 0.175094643 | 0.065587174 | 0.467440998 | 0.000505386 |
| STC2 | 1.822969205 | 1.192936821 | 2.785744108 | 0.005513828 |
| STIM2 | 0.207520358 | 0.068971666 | 0.624382467 | 0.005141971 |
| STK33 | 0.756265153 | 0.593387798 | 0.963850256 | 0.023977102 |
| TACC3 | 1.676519334 | 1.176020947 | 2.39002297 | 0.004287761 |
| TBX2 | 0.509230459 | 0.309201918 | 0.838661229 | 0.008021077 |
| TBX3 | 0.48590878 | 0.287725325 | 0.820599796 | 0.006944539 |
| TCF21 | 0.739787492 | 0.599691021 | 0.91261252 | 0.004898492 |
| TCF3 | 2.916751026 | 1.114118551 | 7.636024497 | 0.029254649 |
| TFCP2 | 0.238649208 | 0.056954637 | 0.999979059 | 0.049996651 |
| TGFA | 1.371949666 | 1.00995929 | 1.863684907 | 0.04303482 |
| TGFB3 | 1.912042416 | 1.038269067 | 3.521154888 | 0.037478939 |
| TGFBR1 | 2.770434726 | 1.214934113 | 6.31746898 | 0.015398179 |
| TGFBR2 | 0.480059574 | 0.243826611 | 0.945168346 | 0.033744265 |
| THBD | 0.583416451 | 0.385010836 | 0.884065393 | 0.011052131 |
| TIMELESS | 1.867611815 | 1.146488905 | 3.042309328 | 0.012105271 |
| TIMP1 | 2.285264892 | 1.363578439 | 3.829948815 | 0.001706594 |
| TLE4 | 0.497465416 | 0.293660078 | 0.842715299 | 0.009424089 |
| TM4SF5 | 1.223738978 | 1.061572141 | 1.410678585 | 0.005373402 |
| TMPRSS4 | 1.257102836 | 1.041058675 | 1.517981241 | 0.017397912 |
| TNFRSF11A | 1.492016883 | 1.044264081 | 2.131754236 | 0.027957974 |
| TNFSF15 | 0.644690542 | 0.473093598 | 0.878527836 | 0.005433296 |
| TPBG | 2.118933971 | 1.358847976 | 3.304182109 | 0.000923926 |
| TRIM16 | 1.53354287 | 1.004221519 | 2.341867494 | 0.047763543 |
| TRIM28 | 2.156425648 | 1.064405523 | 4.368796924 | 0.032905629 |
| TRPM8 | 1.214717157 | 1.041021175 | 1.417394579 | 0.013488921 |
| TSC1 | 0.17133309 | 0.06980161 | 0.420549435 | 0.000117825 |
| TWIST1 | 1.338185222 | 1.094638892 | 1.635918204 | 0.004480578 |
| TXN2 | 2.862277151 | 1.154067543 | 7.098917684 | 0.023258724 |
| TYMS | 1.475956342 | 1.072586501 | 2.031022322 | 0.016839917 |
| UBE3C | 3.434034752 | 1.668599151 | 7.067362268 | 0.000807265 |
| UCHL3 | 2.634960971 | 1.47533273 | 4.706070149 | 0.001059869 |
| UCP2 | 1.801297506 | 1.064153554 | 3.049064389 | 0.02841478 |
| UHRF1 | 1.445908469 | 1.10809252 | 1.886711859 | 0.006608237 |
| USMG5 | 4.760501636 | 1.409822267 | 16.07463321 | 0.011965561 |
| VANGL1 | 6.404773137 | 2.950289154 | 13.90410119 | 2.66E-06 |
| VDR | 2.042112113 | 1.167903693 | 3.570689868 | 0.012266545 |
| VEGFA | 2.380207459 | 1.499417909 | 3.778391278 | 0.000235043 |
| VGLL4 | 3.211281468 | 1.089864764 | 9.462025939 | 0.034341116 |
| WASF3 | 0.608336452 | 0.425840839 | 0.869041212 | 0.006308329 |
| WNT11 | 0.729150922 | 0.605033526 | 0.878729928 | 0.000907091 |
| WT1 | 1.239185692 | 1.057668432 | 1.451854979 | 0.007959979 |
| YWHAZ | 4.859360864 | 1.610543554 | 14.66175066 | 0.005019508 |
